# Ostéo-arthrites tuberculeuses inhabituelles multifocales chez une patiente immunocompétente

**DOI:** 10.11604/pamj.2015.20.212.6015

**Published:** 2015-03-09

**Authors:** Samba Koné, Mariam Gbané-Koné, Abdoulaye Bana, Stanislas André Touré, Adelaide Natacha Kouassi, Akué Gérard Koffi, Edmond Eti, N‘zue Marcel Kouakou

**Affiliations:** 1 Service de Traumato-Orthopédie CHU de Cocody, Abidjan, Côte d'Ivoire; 2 Service de Rhumatologie CHU de Cocody, Abidjan, Côte d'Ivoire

**Keywords:** ostéo-arthrites, tuberculeuses, inhabituelles, osteoarthritis, tuberculosis, unusual

## Abstract

Les formes multifocales de la tuberculose, surviennent habituellement chez des sujets immunodéprimés. Dans les formes multifocales, certaines localisations osseuses sont rares. Les auteurs rapportent le cas d'une patiente de 58 ans, immunocompétente qui présentait une tuberculose multifocale associant une atteinte pulmonaire et des localisations osseuses et articulaires inhabituelles (l’épaule, la cheville et le pied homolatéral, la branche illio-pubienne). Le diagnostic a été histologique (biopsie ostéo-articulaire) et bactériologique (mise en évidence des BAAR dans les crachats). Le traitement a été médico-chirurgical.

## Introduction

La tuberculose multifocale (TM), se définit comme étant l′atteinte de deux sites extra-pulmonaires associée ou non à une atteinte pulmonaire. Elle est rare et grave avec une mortalité élevée (16 à 25% des cas) [1]. Les atteintes diffuses de la tuberculose représentent 09 à 10% des cas et surviennent le plus souvent chez les patients surtout atteints du VIH [1, 2]. Nous rapportons un cas de TM associant une atteinte pulmonaire à 4 localisations osseuses inhabituelles survenues sur un terrain non immunodéprimé.

## Patient et observation

**Figure 1:**
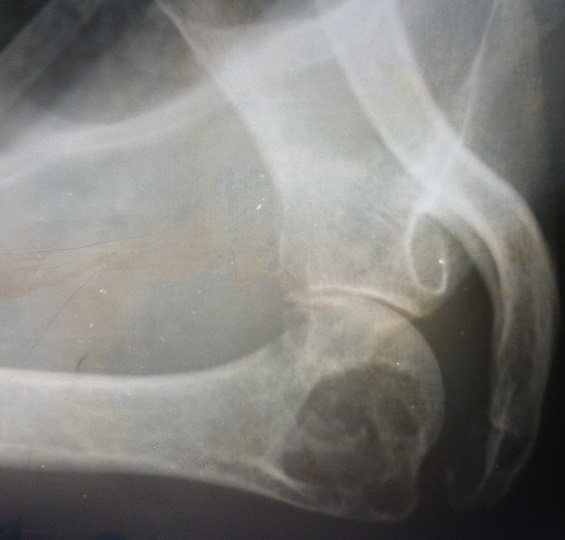
Radiographie de l’épaule gauche de face: pincement de l'interligne, aspect hétérogène, ostéolyse, géodes, de la tête humérale

**Figure 2:**
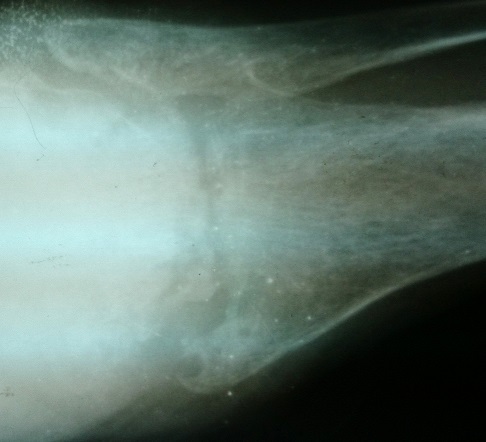
Radiographie de la cheville droite (face) aspect hétérogène de la talo-crurale, pincement de l'interligne, géodes, ostéolyse du dome astragalien et de la malléole interne

**Figure 3:**
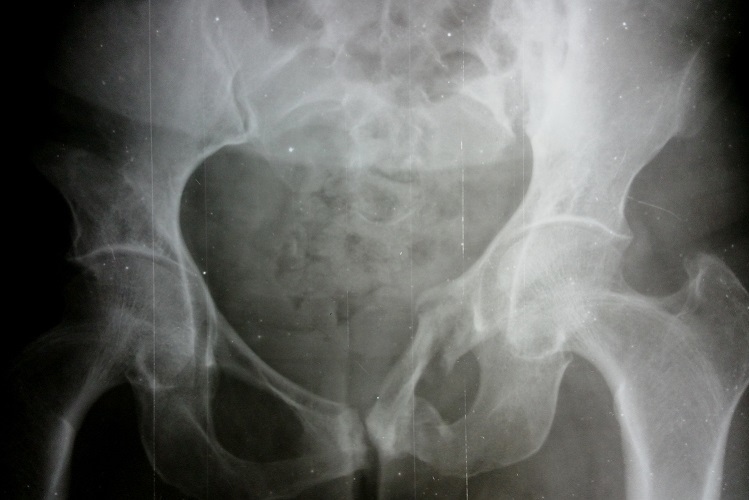
Radiographie du bassin (face), ostéolyse de la branche ilio-pubienne gauche

**Figure 4:**
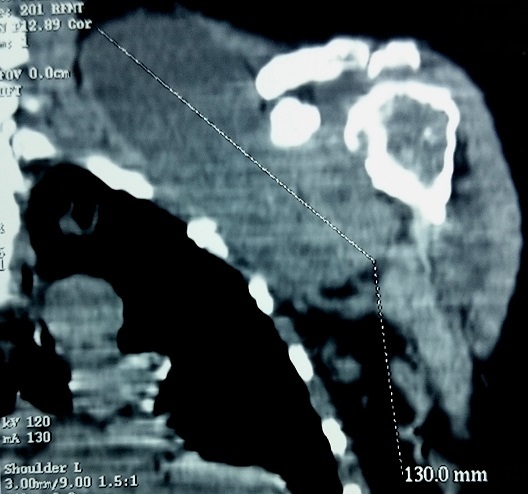
Aspect TDM épaule: coupe coronale: érosion de la tête humérale associée à une volumineuse collection cloisonnée

**Figure 5:**
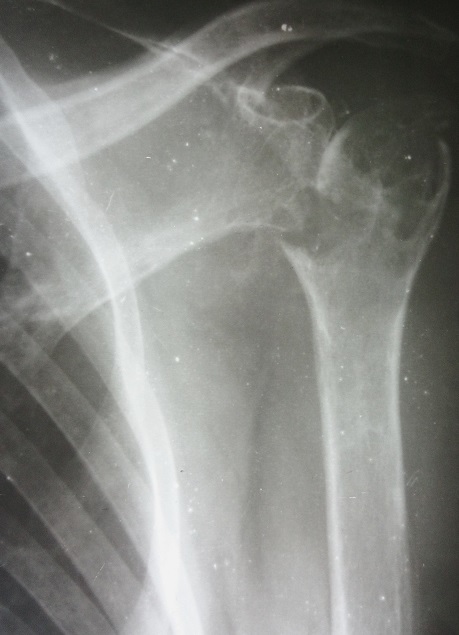
Radiographie de l’épaule de face, image séquellaire d'ostéonécrose septique de la tête humérale

**Figure 6:**
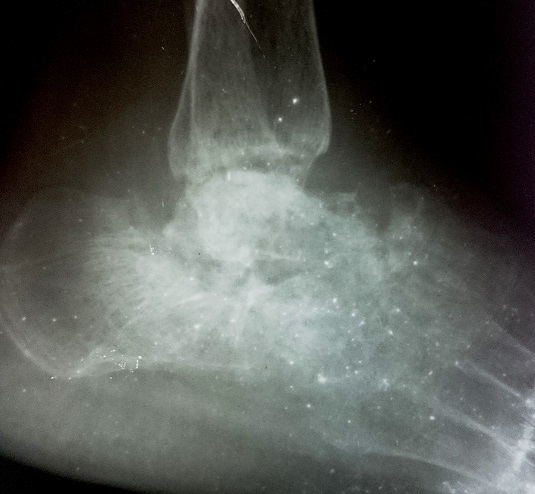
Radiographie de la cheville de face (à 06 mois) image séquellaire, ankylose

Une patiente de 58 ans adressée par le service rhumatologie pour une oligoarthrite chronique de l’épaule gauche et de la cheville droite évoluant depuis 5 mois. De l'anamnèse, il ressortait que cette patiente a consulté pour une tuméfaction douloureuse de l’épaule gauche et de la cheville droite avec une boiterie à la marche. Par ailleurs, il s'y associait une toux sèche et des signes d'imprégnation tuberculeuse. La patiente n'avait pas de notion de contage tuberculeux, ni d'antécédent de tuberculose pulmonaire. Elle n’était pas diabétique. Le bilan articulaire, mettait en évidence une oligoarthrite de l’épaule gauche et de la cheville droite. Ailleurs, l'examen physique avait mis en évidence un syndrome de condensation pulmonaire bilaterale, une douleur exquise au niveau de la symphyse pubienne et des adénopathies inguinales gauches d'environ 1 cm de diamètre, fermes, mobiles et indolores. En rhumatologie les investigations avaient revélé une anémie à 8,8g/dl hypochrome microcytaire, une thrombocytose à 617000 plaquettes, un syndrome inflammatoire avec une CRP à 72 mg et une VS à 78 mm à la première heure. La sérologie rétrovirale était négative. L'IDR à la tuberculine était phlycténullaire. L'examen direct des crachats était revenu fortement positif sur 2 prélèvements (80 puis 70 BAAR/champ); la PCR BK était négative. La radiographie pulmonaire a objectivé un syndrome intersticiel bilatéral. Les radiographies d’épaule (Figure 1), de la cheville (Figure 2) et de la branche illio-pubienne gauche (Figure 3) mettaient en évidence des images d'ostéoarthrite. L’échographie abdominale ne montrait pas d'adénopathie profonde. Le diagnostic de TM fut retenu et la patiente a été mise sous traitement antituberculeux selon notre protocole classique. Malgré 20 jours de chimiothérerapie antituberculeuse, le fébricule persistait de même que le syndrome inflammatoire biologique. Cela a motivé un avis dans notre service. Au vue de l'aspect TDM de l’épaule gauche (Figure 4), l'indication d'une arthrotomie a été posée pour drainage des collections, séquestrectomie et lavages articulaires. Il a été fait un abord antérieur delto-pectoral pour l’épaule et un double abord médial et latéral pour la cheville. Le foyer pubien n’ a pas été abordé. De multiples prélèvements biopsiques (capsule, séquestres) avaient été réalisés lors de l'intervention. La bactériologie s’était avérée négative. L’ examen histologique des prélèvements biopsiques, a mis en évidence un granulome épithéliogiganto cellulaire avec nécrose caséeuse centrale. Un appareillage d'immobilisation d’épaule et de la cheville a été mis en place pendant 15 jours associé à une rééducation fonctionnelle. En postopératoire immediat, on notait une apyrexie franche et une regression significative du syndrome inflammatoire. La polychimoithérapie antituberculeuse a donc été poursuivie pendant un an. A 03 mois d’évolution, le suivi médical par la rhumatologie ne rapportait aucun signe d'intolérance ou de résistance aux anti tuberculeux. Au contrôle clinique à 6 mois on notait au plan fonctionnel une raideur de l’épaule gauche avec score de Constant mauvais, une raideur de la cheville droite. Le bilan radiographique notait des lésions séquellaires post infectieuses (Figure 5, Figure 6) avec une ankylose de la cheville droite.

## Discussion

L'atteinte de l’épaule est rare dans la tuberculose, elle représente 1-10,5% des formes osseuses [3] et toucherait le plus souvent les personnes âgées [2]. Les tuberculoses de la cheville et du pied sont également rares. Elles représentent 6 à 8% des localisations osseuses [2, 4], elles se par une impotence douloureuse de type mécanique avec parfois des fistules spontanées étant donné le caractère superficiel de l'articulation [2]. La tuberculose des branches pubiennes est rare, elle est souvent secondaire à une ostéoarthrite de la symphyse pubienne [5] qui elle même, est très rare moins d'1% des atteintes osseuses [6, 7]. L'originalité de cette observation, c'est l'association chez la même patiente des localisations osseuses inhabituelles. L'ostéite tuberculeuse, représente 10 à 19% des atteintes ostéoarticulaires. Tous les os peuvent être touchés, mais l′affection porte surtout sur les os longs (80%), et la lésion du grand trochanter est la plus fréquente [2]. L'infection à VIH et la corticothérapie au long cours [7] sont citées comme des facteurs favorisants de la TM. Notre patiente étant immunocompétente, nous pensons que le retard de consultation explique la multiplicité des atteintes et la gravité des lésions ostéoarticulaires, comme l'attestent certains auteurs [7]. D'autre part il s'agit vraisemblablement d'atteintes secondaires multiples. Le foyer primitif serait alors pulmonaire au vue des résultats de la bacilloscopie. Le diagnostic de certitude des atteintes extra-pulmonaires repose surtout sur les examens anatomopathologiques des prélèvements [1, 7]. Le diagnostic de certitude des atteintes ostéoarticulaires chez notre patient, a été fait grâce à l'histologie des prélèvements biopsiques. L'arthrotomie précoce devrait etre de règle dans ces localisations articulaires abcédées. Elle permet le drainage d'abcès des parties molles, le débridement ostéo-articulaire avec excision de tous les tissus nécrosés (exérèse des séquestres osseux, curetage osseux), et souvent une synovectomie est nécessaire [8]. L'arthrotomie présente de nombreux avantages: elle permet le diagnostic de certitude grâce aux différents prélèvements, elle préserve l'avenir de la fonction articulaire et enfin elle participe au contrôle de l'infection en association avec la chimiothérapie antituberculeuse. Comme en témoigne l'apyrexie après notre geste chirurgical. L'usage d'appareillage d'immobilisation provisoire en position fonctionnelle, en association à la rééducation sont également indispensables. En présence séquelles fonctionnelles invalidantes (raideur douloureuse, déformation et/ou instabilité) une chirurgie à visée fonctionnelle pourra être réalisée[8]. Cette chirurgie vise la stabilisation ou la reconstruction articulaire par le biais d'arthrodèse ou de remplacement prothétique. Cependant la mise en place d′une prothèse nécessite le respect d′une période de quiescence suffisante et doit être encadrée par la reprise d′une antibiothérapie antituberculeuse.

## Conclusion

La tuberculose multifocale ostéo-articulaire extravertébrale est une forme rare pouvant toucher même des sujets immunocompétents. De la précocité du diagnostic et de la rapidité de mise en route de la chimiothérapie dépendent le pronostic fonctionnel. Une coordination efficace pluridisciplinaire (chirurgien-rhumatologue, bactériologiste, histologiste) est indispensable afin d’éviter tout retard de diagnostic. L'histologie des pièces de biopsie chirurgicale reste l'examen de choix. Le traitement est médico-chirurgical.
